# Exposure to polycyclic aromatic hydrocarbons and bone mineral density in children and adolescents: results from the 2011–2016 National Health and Nutrition Examination Survey

**DOI:** 10.3389/fpubh.2025.1428772

**Published:** 2025-04-17

**Authors:** Peng Zhang, Shuailei Li, Hao Zeng, Yongqiang Sun

**Affiliations:** ^1^Department of Orthopedics, Huaihe Hospital Affiliated to Henan University, Kaifeng, Henan, China; ^2^Henan Luoyang Orthopedic Hospital (Henan Provincial Orthopedic Hospital), Zhengzhou, Henan, China; ^3^Department of Medical Insurance, Huaihe Hospital Affiliated to Henan University, Kaifeng, Henan, China

**Keywords:** polycyclic aromatic hydrocarbons, bone mineral density, children and adolescents, Bayesian kernel machine regression, quantile g-computation

## Abstract

**Introduction:**

Identifying factors that hinder bone development in children and adolescents is crucial for preventing osteoporosis. Exposure to polycyclic aromatic hydrocarbons (PAHs) has been linked to reduced bone mineral density (BMD), although available data, especially in children and adolescents, are limited. We examined the associations between urinary hydroxylated-PAHs (OH-PAHs) and lumbar spine BMD, pelvic BMD, and total BMD among 8–19 years participants (*N* = 1,332) of the 2011–2016 National Health and Nutrition Examination Survey.

**Methods:**

Weighted linear regressions were employed to assess the associations between urinary OH-PAHs and BMD. Additionally, Bayesian kernel machine regression (BKMR) and quantile g-computation (Qgcomp) models were utilized to investigate the effect of co-exposure of PAHs on BMD.

**Results:**

Several urinary OH-PAHs exhibited negative associations with lumbar spine BMD, pelvic BMD, and total BMD in children and adolescents. For instance, an increase of one unit in the natural log-transformed levels of urinary 1-hydroxypyrene and 2&3-Hydroxyphenanthrene was linked with a decrease of −0.014 g/cm^2^ (95% CI: −0.026, −0.002) and −0.018 g/cm^2^ (95% CI: −0.032, −0.004) in lumbar spine BMD, a decrease of −0.021 g/cm^2^ (95% CI: −0.039, −0.003) and −0.017 g/cm^2^ (95% CI: −0.033, −0.001) in pelvic BMD, and a decrease of −0.013 g/cm^2^ (95% CI: −0.023, −0.002) and −0.016 g/cm^2^ (95% CI: −0.026, −0.006) in total BMD. The body mass index modified the associations between urinary OH-PAHs and BMD, revealing negative effects on BMD primarily significant in overweight/obese individuals but not significant in underweight/normal individuals. Both the BKMR model and the Qgcomp model indicated a significant negative correlation between the overall effects of seven urinary OH-PAHs and lumbar spine BMD, pelvic BMD, and total BMD.

**Conclusion:**

Our findings revealed that exposure to PAHs might hinder bone development in children and adolescents, potentially impacting peak bone mass—an essential factor influencing lifelong skeletal health.

## Graphical abstract

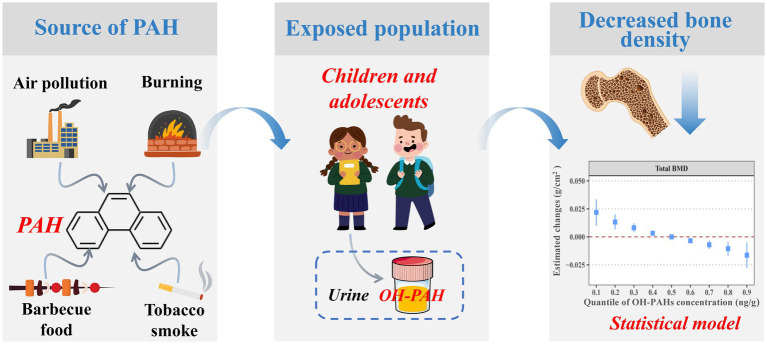

## 1 Introduction

Osteoporosis is a significant public health concern, with approximately 200 million adults worldwide affected by this condition (1). Osteoporosis leads to an increased incidence of bone fractures and mortality, imposing a substantial burden on families and society due to associated medical and caregiving costs. Skeletal accumulation occurs during childhood and adolescence, with peak bone mass in adolescence being a critical factor in the development of osteoporosis in later life (2). Approximately 90% of peak bone mass is attained by age 18–20, and failure to achieve optimal bone accrual during this window increases lifelong osteoporosis risk (3). Adolescents exhibit heightened susceptibility to environmental toxicants due to ongoing bone remodeling, rapid growth rates, and immature detoxification systems (4, 5). Identifying and addressing risk factors that lead to inadequate bone mass accumulation during childhood and adolescence is of paramount importance for osteoporosis prevention. Nevertheless, there has been limited research analyzing the impact of environmental factors on childhood and adolescence bone accrual.

Polycyclic aromatic hydrocarbons (PAHs) are a class of widely distributed environmental pollutants, and human exposure to PAHs can occur through various routes, including inhalation, skin contact, and ingestion (6). Children face unique exposure risks due to higher respiratory rates, and prolonged outdoor activities and proportionally higher intake of contaminated food (7). Emerging evidence suggests that urinary 1-hydroxypyrene (1-OHPyr), a key PAH metabolite, is 30% higher in children aged 6–11 compared to non-smoking adults under similar environmental conditions (8). A systematic review and meta-analysis of 40 studies involving 12,697 children and adolescents further corroborates this finding, demonstrating that urinary 1-OHPyr levels in pediatric populations are consistently elevated compared to non-occupational adults who do not smoke (9). The exposure to PAH is not only related to individual susceptibility and behavioral patterns, but also significantly influenced by geographic heterogeneity and pollution source distribution characteristics (10). Multiple studies have shown that the risk of PAH exposure is significantly increased in industrial intensive areas, transportation corridors, and during the winter heating season (11–13). For instance, monitoring in Rome detected 2.5-fold higher PAH levels in city centers compared to suburbs during heating seasons (13), while industrial cities like Slavonski Brod exhibited PAH concentrations 40% higher than background urban areas (11).

PAHs may affect bone mineral density (BMD) through multiple mechanisms. Experimental studies demonstrate that benzo [a] pyrene disrupts bone homeostasis through aryl hydrocarbon receptor (AhR) activation, suppressing osteoblast differentiation via ERK/MAPK pathway hyperphosphorylation (14). Additionally, PAHs can activate AhR pathways to accelerate osteoclast genesis or disrupt estrogen signaling, potentially altering bone homeostasis more profoundly in growing skeletons (15, 16). This is particularly concerning given that pediatric bone turnover rates are higher than adults (9), potentially amplifying toxicant impacts. Two previous studies have suggested an association between higher urinary hydroxylated-PAH (OH-PAH) concentrations in specific gender adult populations and lower BMD in different skeletal sites (17, 18).

However, few studies have examined PAH effects during the critical bone accrual window of 8–19 years. Current evidence gaps are threefold: (1) Limited data on dose–response relationships in pediatric populations; (2) Insufficient understanding of the unique susceptibility of developing skeletons to PAH exposure; and (3) Lack of analysis of combined exposure effects from multiple PAH congeners. Using a nationally representative sample from NHANES, this study investigates PAH-BMD associations in U.S. adolescents while addressing these knowledge gaps.

## 2 Materials and methods

### 2.1 Study design and population

National Health and Nutrition Examination Survey (NHANES) utilized a sophisticated multi-stage sampling weight design to ensure the selection of a representative sample from the non-institutionalized civilian population of the United States. The survey was overseen by the National Center for Health Statistics (NCHS), and the data from questionnaires, laboratory tests, and physical examinations were made publicly available every 2 years.^1^ Participants in the survey provided written informed consent, and the research protocol was approved by the NCHS Research Ethics Review Board.

**Figure 1:**
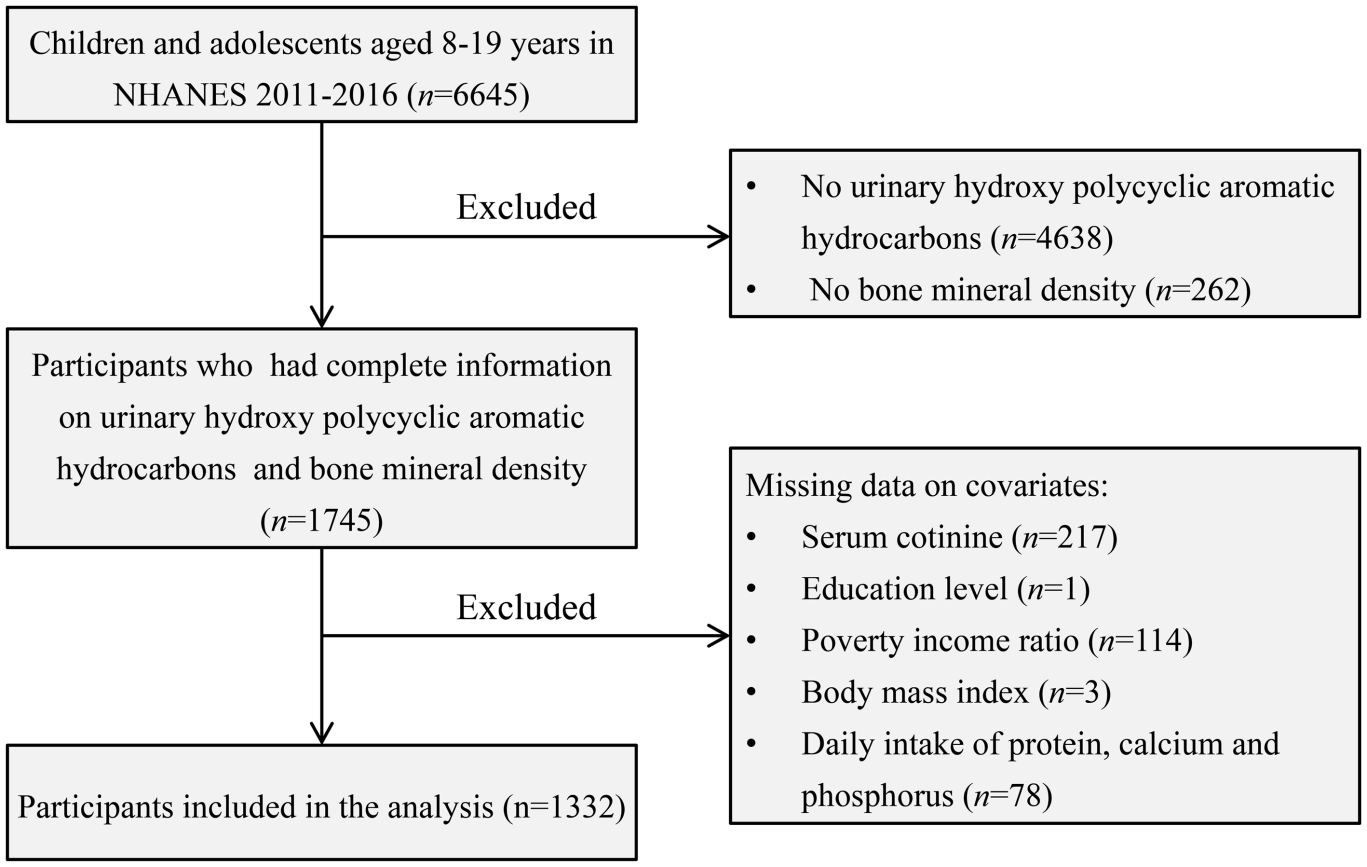
Flow chart of study participant selection process.

Our study focused on children and adolescents aged 8–19, utilizing NHANES data spanning the years 2011–2016. Participants lacking data on BMD, urinary OH-PAHs, and relevant covariates were excluded from the analysis. Ultimately, a total of 1,332 subjects met the inclusion criteria for the final analysis. For a visual representation of the screening process, please refer to Figure 1.

### 2.2 Urinary OH-PAHs and BMD

Spot urine samples were collected from participants during their appointments at the NHANES Mobile Examination Center (MEC). These urine samples underwent processing, storage, and were subsequently shipped to the National Center for Environmental Health for analysis. The analysis included the measurement of seven urinary OH-PAHs, namely 1-Hydroxynaphthalene (1-OHNap), 2-Hydroxynaphthalene (2-OHNap), 3-Hydroxyfluorene (3-OHFlu), 2-Hydroxyfluorene (2-OHFlu), 1-Hydroxyphenanthrene (1-OHPhe), 1-Hydroxypyrene (1-OHPyr), and 2&3-Hydroxyphenanthrene (2&3-OHPhe). These measurements were carried out using isotope dilution high-performance liquid chromatography–tandem mass spectrometry. The detailed laboratory protocols can refer to the NHANES website.^2^ When the concentrations of urinary metals fell below the limit of detection (LOD), they were replaced by LOD divided by the square root of 2. Additionally, the urinary concentrations of OH-PAHs in the study participants were adjusted for corresponding creatinine concentrations and expressed in units of ng/g.

In our study, we assessed three primary outcome variables: lumbar spine BMD, pelvic BMD, and total BMD (19). These measurements were conducted using Dual-Energy X-ray Absorptiometry (DXA) scans, administered by radiology technologists who were both trained and certified. Whole-body scans were performed using Hologic densitometers (Hologic, Inc., Bedford, Massachusetts). For a more comprehensive description of the DXA examination protocol, please consult the Body Composition Procedures Manual available on the NHANES website.^3^

### 2.3 Covariates

The covariates considered in our study, drawn from prior research (18, 20), encompassed demographic factors such as age, gender, race, and body mass index (BMI), poverty income ratio categories (<1, 1–3, >3) (21), education level (below junior school, junior school, high school or above), daily protein intake, daily calcium intake, daily phosphorus intake and serum cotinine. The information about the protein, calcium and phosphorus intake was derived from averaging the first and second 24 h dietary recall data. In instances where the second 24 h recall was unavailable, data from the first recall were utilized. Serum cotinine levels were categorized into high and low levels based on the median value (0.033 ng/mL) among study participants (22).

### 2.4 Statistical analysis

Following NHANES analytic guidelines, we incorporated 6-year sample weights to ensure that our results are representative of the national population aged 8–19 years and to correctly account for the complex, multistage sampling design of NHANES. To compare characteristics between genders, we used design-based methods appropriate for complex survey data: weighted chi-square tests for categorical variables (e.g., race, education) and weighted linear regression for continuous variables (e.g., age, BMI) (18, 23). For skewed urinary OH-PAH concentrations, natural log transformation was applied. Weighted multivariable linear regression models were then used to evaluate associations between log-transformed OH-PAH levels and BMD, adjusting for covariates. Results are reported as estimated BMD changes per unit increase in log-transformed OH-PAHs with 95% confidence intervals (CIs). Additionally, we employed restricted cubic splines regressions with three knots to explore potential non-linear relationships between urinary OH-PAH levels and BMD. We used three knots placed at the 10th, 50th, and 90th percentiles of each OH-PAH exposure distribution, following standard recommendations for restricted cubic splines that balance flexibility and stability. This approach allows for adequate flexibility to detect non-linear relationships while avoiding overfitting (24).

We evaluated potential modification effects of gender (male vs. female), age group (8–13 years vs. 14–19 years), and BMI (underweight/normal vs. overweight/obesity). This assessment involved estimating stratum-specific associations within each subgroup and introducing a multiplicative interaction term in the regression models. An interaction term with a *p*-value below 0.15 was considered statistically significant. The categorization of BMI was based on age-and sex-specific percentiles outlined in the Centers for Disease Control and Prevention growth charts (25).

Given the simultaneous exposure to multiple PAHs and their impact on BMD, we applied two statistical models—Bayesian kernel machine regression (BKMR) and quantile g-computation (Qgcomp) models—to explore the relationship between urinary OH-PAH mixtures and BMD.

(1) The BKMR model enables flexible assessment of the multivariable exposure-response function through kernel functions, allowing for non-linear and non-additive relationships between exposure and response (26). Considering the high correlation among urinary OH-PAHs, as illustrated in Supplementary Figure S1, all urinary OH-PAHs showed significant correlation (*p* < 0.05), with strong correlations observed among 3-OHFlu, 2-OHFlu, 1-OHPhe, 1-OHPyr, and 2&3-OHPhe (correlation coefficient ≥ 0.79). We employed a hierarchical variable selection method to construct the BKMR model for the urinary OH-PAHs mixture based on the magnitude of correlation among them. Urinary OH-PAHs were divided into three groups: Group 1 included 1-OHNap, Group 2 comprised 2-OHNap, and Group 3 encompassed 3-OHFlu, 2-OHFlu, 1-OHPhe, 1-OHPyr, and 2&3-OHPhe. The expression of the BKMR model is as follows:
$$\begin{array}{l} Y_{i}={\mathrm{h[Group}}_{1}=1-{\mathrm{OHNap}}_{i}{\mathrm{,Group}}_{2}=2-{\mathrm{OHNap}}_{i}{\mathrm{,Group}}_{3}\\ \;=(3-{\mathrm{OHFlu}}_{i},2-{\mathrm{OHFlu}}_{i},1-{\mathrm{OHPhe}}_{i},1-{\mathrm{OHPyr}}_{i},\\ \;2\&3-{\mathrm{OHPhe}}_{i})]\;+\beta^{T}Z_{i}+e_{i} \end{array}$$

Where i corresponds to each participant. Y_i_ represents individual BMD. h () signifies the unknown exposure-response function. β denotes the estimated effects of all covariates Z_i_. e_i_ indicates residuals. We used a Gaussian kernel function to model the exposure-response function, capable of handling high-dimensional parameter spaces, employing a Markov Chain Monte Carlo (MCMC) algorithm for 25,000 iterations. The BKMR analysis yielded the following results: (1) Overall OH-PAHs mixture effects on BMD, (2) Individual OH-PAH effects on BMD. For a detailed description, refer to previous researcher (26).

(2) The Qgcomp model is a parametric statistical method that merges weighted quantile sum regression and g-computation, allowing for the assessment of the effects of exposure mixtures (27). This method involves converting all exposure variables into quartiles and then fitting a linear model incorporating exposure, covariates, and outcomes. The aggregate effect of the exposure mixture is quantified as the estimated changes in outcomes corresponding to a quartile increase for all exposure variables. Additionally, each exposure variable is assigned a weight, indicating the significance of the association of each variable in either a positive or negative direction. Notably, the strength of Qgcomp model lies in its ability to avoid the assumption of directional homogeneity. This method allows for the computation of both positive and negative associations between individual exposure variables and outcomes. The detailed description referred to the previous study (27).

Sensitivity analysis comprised two key components: (1) Exclusion of individuals with abnormal creatinine values (<30 mg/dL or >300 mg/dL) was conducted to validate the association between urinary OH-PAHs and BMD (28). (2) An extended approach utilizing the Qgcompint model, which is an extension of the Qgcomp package, was employed to evaluate potential effect measure modifications of the overall mixture effect. This model incorporates interaction terms between the OH-PAH mixture and covariates such as gender, age group, and BMI to assess how these factors modify the effect of PAH exposure on BMD (29). (3) Given that 1-OHPyr had a detection rate of 85%, we conducted a secondary analysis excluding participants whose 1-OHPyr measurement fell below the detection limit.

All data analyses were performed with R (4.3.1). Except for the interaction *p*-value, all other significance levels were set at 0.05. Weighted linear regression, restricted cubic splines regression, bmkr, Qgcomp, and Qgcompint were implemented by R packages “survey,” “rms,” “bkmr,” “qgcmop,” and “Qgcompint,” respectively.

## 3 Results

### 3.1 Characteristics of participants

As shown in Table 1, this study involved a total of 1,332 participants, including 690 males and 642 females. Their weighted average age and BMI were 13.6 years and 22.9 kg/m^2^, respectively. The majority of the study subjects were non-Hispanic white, had high school or above education, and had a poverty income ratio of 1–3. Among males, the intake of serum cotinine, protein, calcium, and phosphorus was significantly higher compared to females (*p* < 0.05).

**Table 1:** Weighted characteristics of participants (*N* = 1,332).

Characteristics	Total	Male	Female	*p*
*N* ^a^	1,332	690	642	
Age, years	13.6 ± 3.30	13.5 ± 3.39	13.8 ± 3.21	0.283
BMI, kg/m^2^	22.9 ± 6.31	22.4 ± 5.93	23.4 ± 6.68	0.114
Race, *n* (%)				0.259
Non-Hispanic White	726 (54.5%)	367 (53.2%)	359 (55.9%)	
Hispanic	304 (22.8%)	158 (23.0%)	146 (22.7%)	
Non-Hispanic Black	173 (13.0%)	100 (14.4%)	73 (11.4%)	
Other Race	129 (9.7%)	65 (9.4%)	64 (10.0%)	
Education level, *n* (%)				0.537
Below junior school	419 (31.5%)	224 (32.4%)	195 (30.3%)	
Junior school	397 (29.8%)	211 (30.6%)	187 (29.1%)	
High school or above	516 (38.7%)	255 (37.0%)	261 (40.6%)	
Poverty income ratio, *n* (%)				0.300
<1	310 (23.3%)	149 (21.6%)	161 (25.1%)	
1–3	555 (41.6%)	285 (41.3%)	269 (41.9%)	
>3	467 (35.1%)	256 (37.1%)	212 (33.0%)	
Cotinine, *n* (%)				0.017
Low	680 (51.0%)	327 (47.4%)	353 (55.0%)	
High	652 (49.0%)	363 (52.6%)	289 (45.0%)	
Protein, g	74.3 ± 32.7	84.1 ± 36.4	63.8 ± 24.2	<0.001
Calcium, g	1.05 ± 0.518	1.17 ± 0.540	0.922 ± 0.459	<0.001
Phosphorus, g	1.34 ± 0.552	1.49 ± 0.593	1.17 ± 0.448	<0.001
Lumbar spine BMD, g/cm^2^	0.888 ± 0.186	0.855 ± 0.191	0.924 ± 0.174	<0.001
Pelvis BMD, g/cm^2^	1.09 ± 0.230	1.07 ± 0.249	1.11 ± 0.205	0.069
Total BMD, g/cm^2^	0.965 ± 0.150	0.969 ± 0.160	0.961 ± 0.138	0.501
1-OHNap, ng/g	886 (497, 1,631)	862 (481, 1,615)	893 (516, 1,693)	0.892
2-OHNap, ng/g	4,253 (2,580, 7,861)	3,813 (2,319, 6,589)	4,898 (2,830, 8,883)	<0.001
3-OHFlu, ng/g	70.5 (44.6, 109)	69.0 (44.2, 106)	71.1 (45.0, 111)	0.570
2-OHFlu, ng/g	156 (111, 245)	153 (107, 244)	163 (117, 246)	0.303
1-OHPhe, ng/g	95.5 (64.7, 142)	90.4 (61.0, 134)	100 (71.7, 153)	0.164
1-OHPyr, ng/g	132 (86.9, 202)	121 (79.8, 196)	142 (100, 211)	0.556
2&3-OHPhe, ng/g	108 (77.9, 155)	107 (74.0, 159)	110 (79.7, 152)	0.023

BMI, Body mass index; BMD, Bone mineral density; 1-OHNap, 1-Hydroxynaphthalene; 2-OHNap, 2-Hydroxynaphthalene; 3-OHFlu, 3-Hydroxyfluorene; 2-OHFlu, 2-Hydroxyfluorene; 1-OHPhe, 1-Hydroxyphenanthrene; 1-OHPyr, 1-Hydroxypyrene; 2&3-OHPhe, 2&3-Hydroxyphenanthrene.

a Unweighted sample number in the group.

The weighted average BMD of the participants was 0.888 g/cm^2^ for lumbar spine BMD, 1.09 g/cm^2^ for pelvic BMD, and 0.965 g/cm^2^ for total BMD. Female participants exhibited significantly higher lumbar spine BMD than males (*p* < 0.05), while there were no significant gender differences in pelvic BMD and total BMD. Supplementary Table S1 provides the descriptive statistics for the seven urinary OH-PAHs measured in our study. Detection frequencies ranged from 85.2% (1-OHPyr) to nearly 100%. The mean concentrations were highest for 2-OHNap and lowest for 3-OHFlu. Except for 2-OHNap and 2&3-OHPhe, the concentrations of other OH-PAHs did not show significant differences between different genders.

### 3.2 Association of urinary OH-PAHs with BMD: weighted linear regression model

**Figure 2:**
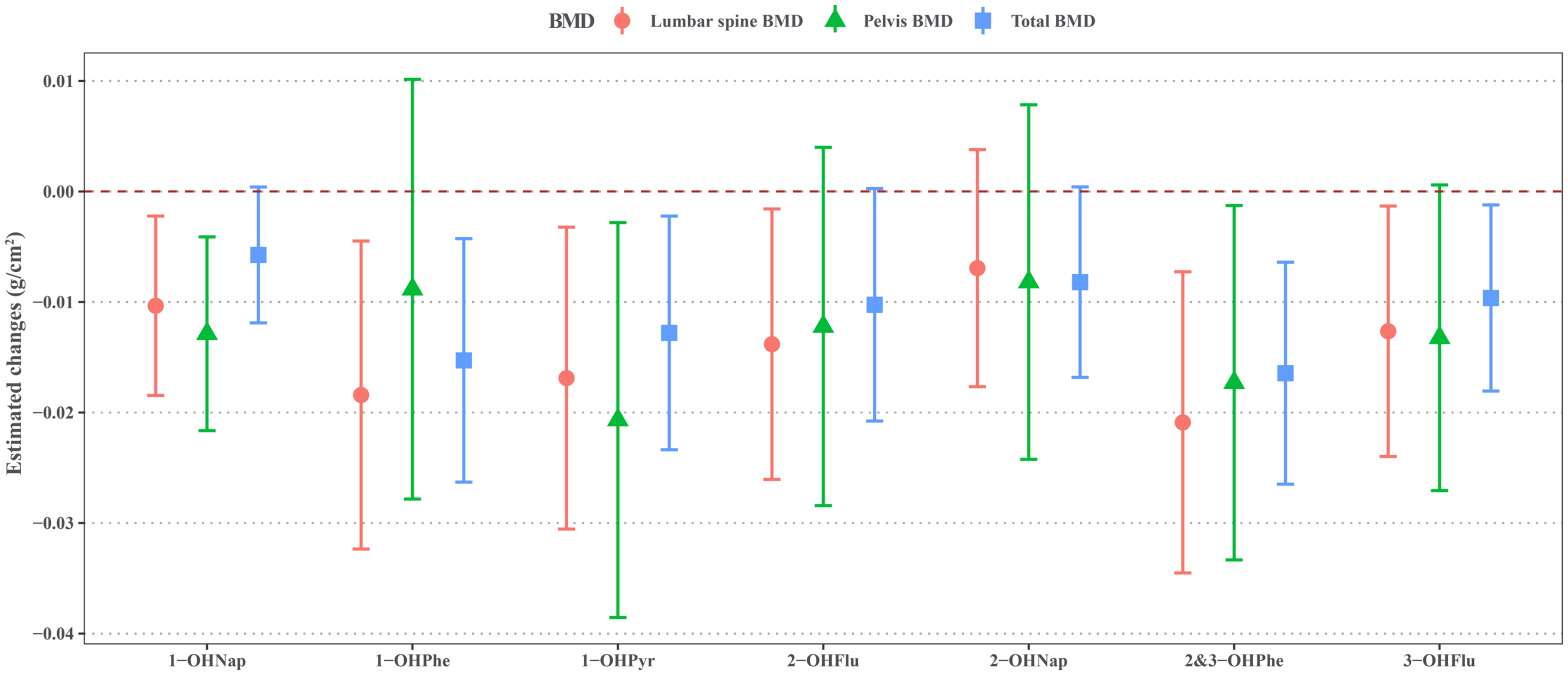
Estimated changes for associations of urinary OH-PAHs with lumbar spine BMD, pelvis BMD, and total BMD after adjusted for gender, age, race, poverty income ratio, education level, BMI, cotinine, and daily protein, calcium, and phosphorus intake. OH-PAHs, hydroxy polycyclic aromatic hydrocarbons; BMD, bone mineral density; BMI, body mass index.

As shown in Figure 2, the majority of OH-PAHs exhibit a significant negative correlation with BMD. Each unit increase of natural log-transformed urinary 1-OHNap, 1-OHPhe, 1-OHPyr, 2-OHFlu, 2&3-OHPhe, and 3-OHFlu was associated with a decrease of −0.010 g/cm^2^ (95% CI: −0.018, −0.002), −0.013 g/cm^2^ (95% CI: −0.024, −0.001), −0.014 g/cm^2^ (95% CI: −0.026, −0.002), −0.021 g/cm^2^ (95% CI: −0.035, −0.007), −0.018 g/cm^2^ (95% CI: −0.032, −0.004), and −0.017 g/cm^2^ (95% CI: −0.031, −0.003) in lumbar spine BMD. Pelvic BMD decreased by −0.013 g/cm^2^ (95% CI: −0.022, −0.004), −0.017 g/cm^2^ (95% CI: −0.033, −0.001), and −0.021 g/cm^2^ (95% CI: −0.039, −0.003) with one unite increase of natural log-transformed urinary 1-OHNap, 2&3-OHPhe, and 1-OHPyr. Total BMD reduced by −0.010 g/cm^2^ (95% CI: −0.018, −0.001), −0.016 g/cm^2^ (95% CI: −0.026, −0.006), −0.015 g/cm^2^ (95% CI: −0.026, −0.004), and −0.013 g/cm^2^ (95% CI: −0.023, −0.002) with per unit increase of natural log-transformed urinary 3-OHFlu, 2&3-OHPhe, 1-OHPhe, and 1-OHPyr.

Supplementary Figures S2–S4 illustrate restricted cubic spline models depicting the nonlinear associations of urinary OH-PAH levels with lumbar spine BMD (Supplementary Figure S2), pelvic BMD (Supplementary Figure S3), and total BMD (Supplementary Figure S4). These plots suggest non-linear relationships between 1-OHPyr and pelvic BMD, as well as between 1-OHPhe and both pelvic BMD and total BMD (*P* for nonlinearity < 0.05).

Supplementary Figures S5, S6, respectively, illustrate subgroup analysis of the associations between urinary OH-PAHs and BMD among different gender (males and females) and age group (< 14 years and ≥ 14 years). Regarding gender, significant interactions between 1-OHPhe and gender for pelvic BMD and total BMD are observed, as well as between 3-OHFlu and gender for total BMD (all *P*-interaction < 0.15). Specifically, the effect of 1-OHPhe on pelvic BMD is not significant in both males and females, and the effect on total BMD is significant only in males, while the effect of 3-OHFlu on total BMD is significant only in females. Concerning age, only the interaction between 2-OHNap and age for the BMD is significant (*P*-interaction < 0.15); the effect of 2-OHNap on lumbar spine BMD and pelvic BMD is not significant in both age groups (<14 years and ≥14 years), while the negative effect on total BMD is significant only in the ≥14 years age group.

**Figure 3:**
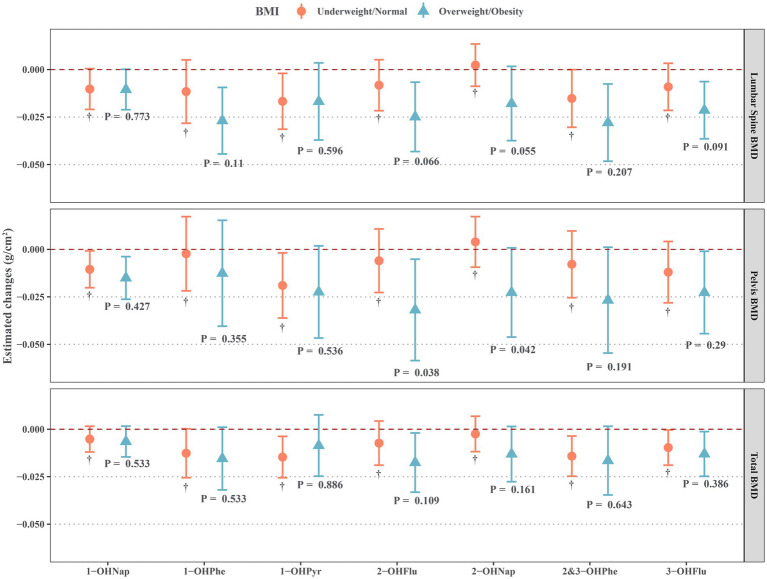
Estimated changes for associations of urinary OH-PAHs with lumbar spine BMD, pelvis BMD, and total BMD in different BMI groups after adjusted for gender, age, race, poverty income ratio, education level, cotinine, and daily protein, calcium, and phosphorus intake. OH-PAHs, hydroxy polycyclic aromatic hydrocarbons; BMD, bone mineral density; BMI, body mass index.

As shown in Figure 3, we also identified significant interactions between some OH-PAHs and BMI (underweight/normal and overweight/obesity) on BMD, such as 1-OHPhe, 2-OHFlu, 2-OHNap, 3-OHFlu, and BMI on lumbar spine BMD, 2-OHFlu and BMI on pelvic BMD, and 2-OHFlu and BMI on total BMD (all *P*-interaction < 0.15). These OH-PAHs exhibited a negative effect on BMD that was significant only in overweight/obesity individuals, while not significant in underweight/normal individuals.

### 3.3 Association of urinary OH-PAHs with BMD: BKMR model

**Figure 4:**
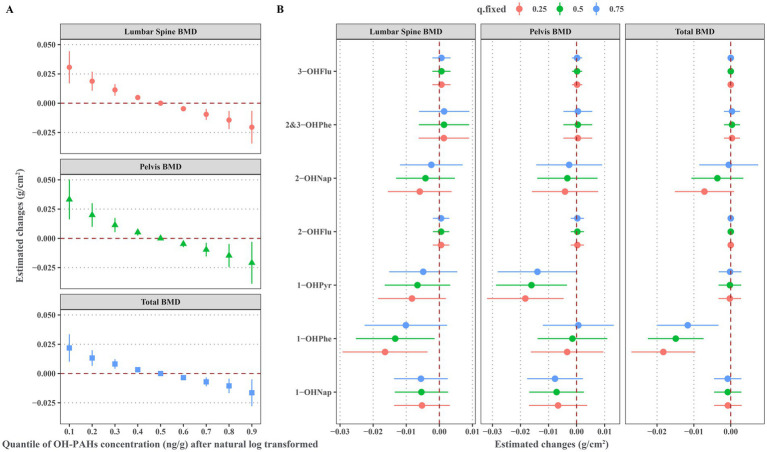
Associations of urinary OH-PAHs with BMD based BMKR model after adjusted for gender, age, race, poverty income ratio, education level, BMI, cotinine, and daily protein, calcium, and phosphorus intake. **(A)** Overall effect of urinary OH-PAHs mixture on BMD through comparing the estimated changes of BMD when urinary OH-PAHs fixing at the 10th-90th percentiles with these when urinary OH-PAHs fixing at the 50th percentiles; **(B)** Effects of individual urinary OH-PAH with BMD through comparing the estimated changes of BMD when each OH-PAH was in the 75th percentile with these in its 25th percentile and when all other urinary OH-PAHs were fixed at the 25th, 50th and 75th percentiles, respectively. OH-PAHs, hydroxy polycyclic aromatic hydrocarbons; BMD, bone mineral density; BMKR, Bayesian kernel machine regression; BMI, body mass index.

As illustrated in Figure 4A, the significant negative linear associations exist between the overall effects of urinary OH-PAHs mixture with lumbar spine BMD, pelvic BMD, and total BMD. Figure 4B displays the associations between individual OH-PAH and BMD in the BMKR model. The results indicate that 1-OHPhe is the primary contributor to the decrease in lumbar spine BMD and total BMD, while 1-OHPyr is the main contributor to the decrease in pelvic BMD. When the concentrations of the other urinary OH-PAHs were held constant at the 50th percentile, each increase of one quartile range in the natural log-transformed of urinary 1-OHPhe was significantly associated with a decrease of −0.013 g/cm^2^ (95% CI: −0.025, −0.001) in lumbar spine BMD and −0.015 g/cm^2^ (95% CI: −0.022, −0.007) in total BMD. Additionally, each increase of one quartile range in the natural log-transformed of urinary 1-OHPyr was significantly associated with a decrease of −0.016 g/cm^2^ (95% CI: −0.029, −0.003) in pelvic BMD.

Supplementary Figure S7 depicts exposure-response curves for the associations between individual OH-PAH and BMD in the BKMR model. These findings confirm significant negative associations between 1-OHPhe and lumbar spine BMD and total BMD, as well as between 1-OHPyr and pelvic BMD.

### 3.4 Association of urinary OH-PAHs with BMD: Qgcomp model

**Figure 5:**
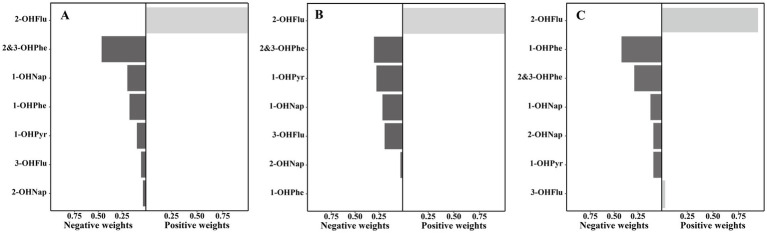
Weights of each urinary OH-PAH in associations with **(A)** lumbar spine BMD, **(B)** pelvis BMD, and **(C)** total BMD based on Qgcomp models after adjusted for gender, age, race, poverty income ratio, education level, BMI, cotinine, and daily protein, calcium, and phosphorus intake. OH-PAHs, hydroxy polycyclic aromatic hydrocarbons; BMD, bone mineral density; Qgcomp, quantile g-computation; BMI, body mass index.

As shown in Supplementary Table S2, the Qgcomp model reveals a significant negative association between the overall effect of the urinary OH-PAHs mixture and BMD. For one quartile range increase in the natural log-transformed of urinary OH-PAHs mixture, lumbar spine BMD, pelvic BMD, and total BMD decreased by −0.012 g/cm^2^ (95% CI: −0.021, −0.004), −0.014 g/cm^2^ (95% CI: −0.025, −0.003), and −0.010 g/cm^2^ (95% CI: −0.016, −0.003), respectively. As shown in Figure 5, 2&3-OHPhe is assigned the largest negative weight to the decrease in lumbar spine BMD (weight = 0.46), while 2&3-OHPhe (weight = 0.30) and 1-OHPyr (weight = 0.28) are the main contributors to the decrease in pelvic BMD. Additionally, 1-OHPyr has the highest negative weight to the decrease in BMD (weight = 0.42).

### 3.5 Sensitivity analysis

Supplementary Figure S8 displays the relationships between urinary OH-PAHs and BMD in subjects with normal creatinine levels (30–300 mg/dL). Except for the significant negative correlation between 1-OHPyr and lumbar and pelvic BMD, which shifted to marginal negative correlation (*p* = 0.059 and *p* = 0.056), the other results are consistent with the main analysis results. Supplementary Figure S9 demonstrates the differences in the overall effects of urinary OH-PAHs mixture on BMD among different genders, age groups, and BMI categories. Similar to the stratified analysis in the main study, we observe significant interactions between urinary OH-PAHs mixture and BMI for lumbar spine BMD (*P*-interaction = 0.05), with the overall negative effect of the mixture being significant only in overweight/obesity individuals, not in those with underweight/normal subjects. After excluding individuals with undetectable 1-OHPyr levels, we identified significant negative associations between urinary 1-OHPyr and BMD (Supplementary Table S3), consistent with our primary analysis results.

## 4 Discussion

To our knowledge, this is the first study to examine the effects of urinary OH-PAHs on BMD among children and adolescents. We found that some urinary OH-PAHs were negatively associated with lumbar spine BMD, pelvic BMD, and total BMD. BMI modified the associations between urinary OH-PAHs and BMD, and some OH-PAHs exhibited negative effects on BMD, only significant in overweight/obesity individuals, while not significant in underweight/normal individuals. In mixture analysis, both the BKMR model and the Qgcomp model found a significant negative correlation between the overall effect of urinary OH-PAHS mixture and lumbar spine BMD, pelvic BMD, and total BMD; urinary 1-OHPyr and 1-OHPhe were identified as the primary contributors to the decrease in pelvic BMD and total BMD, respectively.

Currently, there is a lack of research on the impact of PAHs exposure on BMD in children and adolescents, making it difficult to directly compare our study’s findings with existing literature. There are limited studies that have reported associations between PAHs exposure in adults and BMD, but the results are inconsistent. For instance, Di et al. found a significant negative association between adult urinary 3-OHFlu, 2-OHFlu, and 1-OHPhe levels and lumbar spine BMD in NHANES 2005–2010 and 2013–2014 (*N* = 6,766) (20). Conversely, another study in NHANES 2005–2010 (*N* = 1768) found that adult lumbar spine BMD was significantly negatively associated only with urinary 3-OHPhe at the second tertile, and this study also found a significant positive correlation between urinary 1-OHPyr and trochanteric BMD (18). Additionally, in NHANES 2001–2004 (*N* = 2,987), only female 2-OHPhe levels were significantly negatively correlated with total BMD (17). The disparities in these study results may be attributed to differences in sample size, levels of urinary OH-PAHs, and the skeletal sites. Our study extends the findings from these previous studies, and the discovery of the detrimental effects of PAHs exposure on BMD in children and adolescents holds significant implications for the field of research on the impact of PAHs exposure on bone health in the general population.

Exposure to PAHs, the potential biological mechanisms underlying the decrease in BMD remain unclear, but several pathways have been identified. First, PAH exposure can disrupt bone turnover equilibrium, leading to increased bone resorption (15). In an epidemiological study (30), a significant correlation was found between urinary OH-PAH levels in adults and elevated N-terminal peptide levels (a biomarker reflecting bone resorption). Second, PAHs exhibit estrogen-like effects, reducing the inhibitory action of estrogen on osteoclasts and increasing bone resorption (16). Third, PAHs can induce a pro-inflammatory state in the body, with increased levels of inflammatory mediators such as Tumor Necrosis Factor-*α* and Interleukin-6 (31), which can stimulate the expression of receptor activator of nuclear factor-κB ligand by osteoblasts. This, in turn, activates osteoclasts, leading to a reduction in bone mass (32). Fourth, PAHs can generate a substantial amount of reactive oxygen species by activating aryl hydrocarbon receptors, which can promote apoptosis of mesenchymal stem cells (precursors to osteoblasts), osteoblasts, and osteocytes, thereby reducing bone formation (33).

Our findings indicate a significant negative association between certain urinary OH-PAH levels and BMD, notably in overweight/obese children and adolescents. This suggests increased susceptibility within this demographic, aligning with previous research demonstrating that higher body fat percentage and total fat mass negatively impact lumbar spine BMD and total BMD (34). The mechanisms by which PAHs negatively influence bone density, particularly in the context of obesity, may involve several biological pathways: (1) Altered Metabolism and Lipid Homeostasis: PAHs have been shown to promote preadipocyte differentiation in adipose tissues while potentially disrupting lipid metabolism. Activation of peroxisome proliferator-activated receptors (PPARs) by PAHs may lead to altered adipocyte function, which can adversely affect bone health (35). This dual impact on both fat and bone tissues could exacerbate the negative effects of obesity on BMD. (2) Inflammation and Bone Remodeling: Obesity is associated with chronic low-grade inflammation, which is a known risk factor for bone loss. Adipose tissue secretes pro-inflammatory cytokines that can impair osteoblast function and promote osteoclastogenesis, leading to decreased bone formation and increased bone resorption (36). The presence of PAHs may further exacerbate this inflammatory state, intensifying the negative effects on BMD in overweight/obese individuals. (3) Hormonal Disruption: PAHs are recognized endocrine disruptors that may alter hormonal balance, particularly affecting sex hormones, which play critical roles in bone health. In adolescents, the relative abundance of estrogen and androgens is crucial for achieving peak bone mass (3, 16). Disruption of these hormonal signals by PAHs, combined with the altered hormonal milieu characteristic of obesity, may further contribute to compromised bone health. Previous studies have shown a significant positive correlation between urinary OH-PAH levels and BMI in children and adolescents, underscoring the importance of considering body composition when evaluating the health impacts of environmental exposures (37). This correlation reinforces our findings, suggesting that weight status amplifies the adverse effects of PAHs on bone density.

In real-life situations, the human body is typically exposed to various PAHs simultaneously. This study employed BKMR and Qgcomp to evaluate the joint effects of PAH mixtures on BMD. These models were selected based on their complementary strengths in addressing distinct aspects of environmental mixture analysis. The BKMR model was chosen to account for potential non-linear dose–response relationships and interactions among PAHs, which are biologically plausible given their diverse mechanisms of toxicity. For instance, certain PAHs may disrupt bone homeostasis through AhR activation or oxidative stress pathways, and their combined effects could be synergistic or non-additive (16, 38). BKMR’s semi-parametric framework allows flexible modeling of such complexities without assuming linearity a priority (26). Conversely, the Qgcomp model was applied to quantify the overall linear effect of the PAH mixture while estimating component weights under an additive assumption (27). This parametric approach is advantageous for risk assessment, as it provides interpretable estimates of cumulative effects, which are critical for informing public health interventions. By integrating both approaches, this study balances methodological rigor with practical interpretability, addressing the dual need to explore mechanistic complexity (via BKMR) and quantify actionable risks (via Qgcomp). This strategy aligns with recent methodological frameworks advocating for multi-model analyses in environmental mixtures research (39). Furthermore, both the BKMR and Qgcomp models identified urinary 1-OHPyr and 1-OHPhe as the primary contributors to the decrease in pelvic BMD and total BMD, emphasizing the need for prioritizing the control of these specific PAHs in preventing BMD reduction in children and adolescents. However, for lumbar BMD, the primary contributors identified by the two models differed: BKMR highlighted 1-OHPhe, whereas Qgcomp emphasized 2&3-OHPhe. These metabolites, though derived from the same parent PAH (phenanthrene), may differentially influence bone health due to variations in hydroxylation patterns and bioavailability (40). The discrepancy between models could reflect distinct methodological approaches—BKMR’s incorporation of nonlinearity and interactions versus Qgcomp’s linear additive framework. This suggests both metabolites may contribute to lumbar BMD reduction through complementary pathways, necessitating further toxicological and epidemiological validation.

A critical aspect often overlooked is the potential synergistic or additive effects of PAHs in conjunction with other environmental contaminants. Studies investigating combined exposures have indicated that phenolic compounds, chlorophenol pesticides, and phthalates may exacerbate the detrimental effects of PAHs on bone health. For instance, in a study examining the joint effects of these compounds, results indicated that co-exposure significantly impacted BMD, highlighting the importance of accounting for multiple environmental pollutants when assessing bone health (20). Research has shown that endocrine disruptors, including PAHs, can interact with pathways involved in bone metabolism, further complicating their effects (41). For example, a review highlighted that chronic exposure to endocrine disruptors like phthalates and per-and polyfluoroalkyl substances can lead to alterations in bone remodeling processes, which may compound the adverse effects of PAHs on BMD (42). The interplay between PAHs and these pollutants can create a cumulative burden on bone health, emphasizing the need for comprehensive studies that address multiple exposures to adequately assess risks to BMD. Despite the existing literature linking PAH exposure to impaired bone health, there is still limited research on the co-exposure of PAHs with other toxicants and their collective impact on bone density. Further investigations are warranted to better understand these interactions and their implications for public health.

This study has several notable strengths. First, our use of NHANES data—a nationally representative sample with rigorous protocols—enhances the generalizability of findings to U.S. children and adolescents. Second, we focused on a critical yet understudied developmental window (8–19 years), during which bone mass accrual peaks and environmental insults may exert lifelong consequences. Third, we employed advanced mixture modeling approaches (BKMR and Qgcomp) to evaluate co-exposure effects, addressing a key limitation of single-pollutant studies. Additionally, stratified analyses revealed heightened susceptibility in overweight/obese individuals, highlighting metabolic status as a modifier of PAH toxicity. Our findings underscore the need for targeted interventions to reduce PAH exposure in children and adolescents, contributing to the prevention of future osteoporosis and associated health outcomes.

However, this study also has the following drawbacks. Firstly, the cross-sectional study design cannot establish a causal temporal relationship between PAHs exposure and changes in BMD. Secondly, urinary OH-PAH levels may significantly change over time due to the stochastic nature of exposure and variations in PAH pharmacokinetics (43). Using single-point urine samples to measure individual PAH exposure concentrations may lead to exposure misclassification. Thirdly, this study did not include children under the age of 8 due to the lack of BMD data for this age group. As a result, it was not possible to explore the effect of PAHs exposure on early childhood BMD. Finally, the study focused only on the impact of PAHs exposure on BMD in children and adolescents. However, other toxic substances in the environment, such as per-and polyfluoroalkyl substances, phthalates, and lead, can also contribute to changes in BMD in children and adolescents (20, 44, 45). The study did not account for these potential confounding factors.

## 5 Conclusion

In conclusion, we found that higher levels of PAHs exposure in children and adolescents are associated with decreased BMD, with a potentially greater effect on overweight/obesity individuals. While further validation of our findings is necessary, reducing environmental PAHs exposure during childhood and adolescence may potentially mitigate bone mass loss, thus improving peak BMD and preventing osteoporosis.

## Data Availability

The raw data supporting the conclusions of this article will be made available by the authors, without undue reservation.

## Footnotes

^1^ https://www.cdc.gov/nchs/nhanes/index.htm

^2^ https://wwwn.cdc.gov/nchs/data/nhanes/2015-2016/labmethods/PAH-I-PAHS-I-MET-508.pdf

^3^ https://wwwn.cdc.gov/nchs/data/nhanes/2015-2016/manuals/2016_Body_Composition_Procedures_Manual.pdf
