# Supplementary material for: Exposure to polycyclic aromatic hydrocarbons and bone mineral density in children and adolescents: results from the 2011–2016 National Health and Nutrition Examination Survey
Source: Front Public Health. 2025 Apr 17;13:1428772. doi: 10.3389/fpubh.2025.1428772 (PMC12043670; doi:10.3389/fpubh.2025.1428772)
Supplement: Supplementary file 1 [file Data_Sheet_1.docx]

| **Table S1** Distribution of urinary OH-PAHs (N = 1332) of participants | | | | | | | | |
| --- | --- | --- | --- | --- | --- | --- | --- | --- |
| Urinary OH-PAHs,  ng/L | LOD, ng/L | Detection  Frequency | Mean | Percentile | | | | |
|  |  |  |  | 5th | 25th | 50th | 75th | 95th |
| 1-OHNap | 60 | 99.8% | 2633.9 | 165.6 | 445.8 | 920.0 | 1815.3 | 9453.8 |
| 2-OHNap | 90 | 100.0% | 8317.3 | 697.7 | 2292.3 | 4726.0 | 10486.3 | 26601.2 |
| 3-OHFlu | 8 | 99.2% | 139.4 | 14.6 | 39.0 | 75.0 | 140.3 | 418.9 |
| 2-OHFlu | 8 | 99.8% | 285.6 | 33.6 | 89.0 | 167.0 | 314.3 | 884.9 |
| 1-OHPhe | 9 | 99.2% | 140.2 | 20.0 | 51.0 | 94.0 | 174.0 | 404.5 |
| 1-OHPyr | 70 | 85.2% | 215.2 | 49.5 | 74.0 | 137.5 | 247.0 | 625.8 |
| 2&3-OHPhe | 10 | 99.6% | 175.6 | 22.6 | 62.0 | 116.0 | 206.0 | 487.4 |
| Abbreviations: OH-PAHs, Hydroxy polycyclic aromatic hydrocarbons; 1-OHNap, 1-Hydroxynaphthalene; 2-OHNap,  2-Hydroxynaphthalene; 3-OHFlu, 3-Hydroxyfluorene; 2-OHFlu, 2-Hydroxyfluorene; 1-OHPhe, 1-Hydroxyphenanthrene;  1-OHPyr, 1-Hydroxypyrene; 2&3-OHPhe, 2&3-Hydroxyphenanthrene; LOD, Limit of detection. | | | | | | | | |


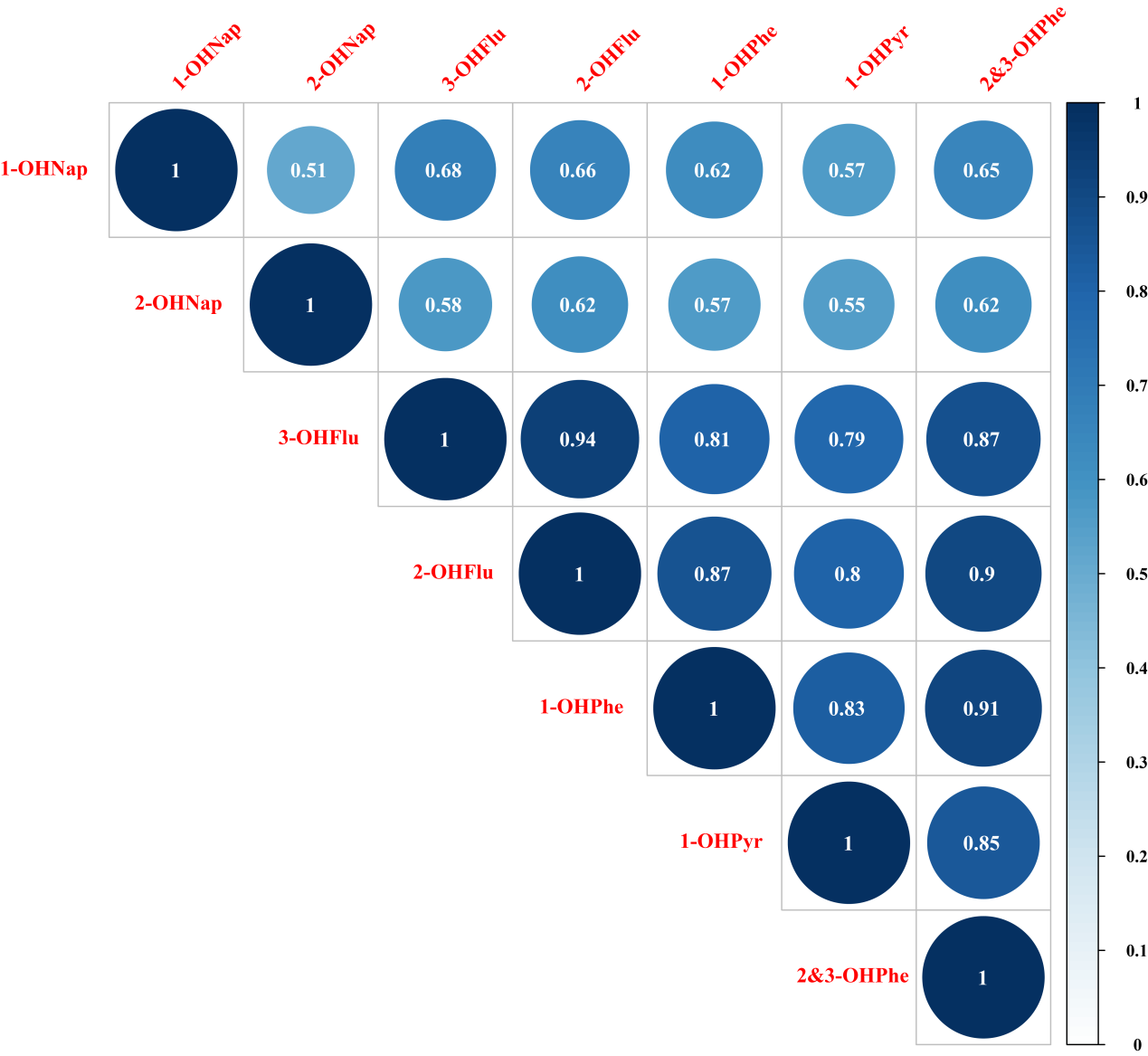


**Fig. S1** The correlation heatmap for urinary OH-PAHs. Abbreviations: OH-PAHs, Hydroxy polycyclic aromatic hydrocarbons.


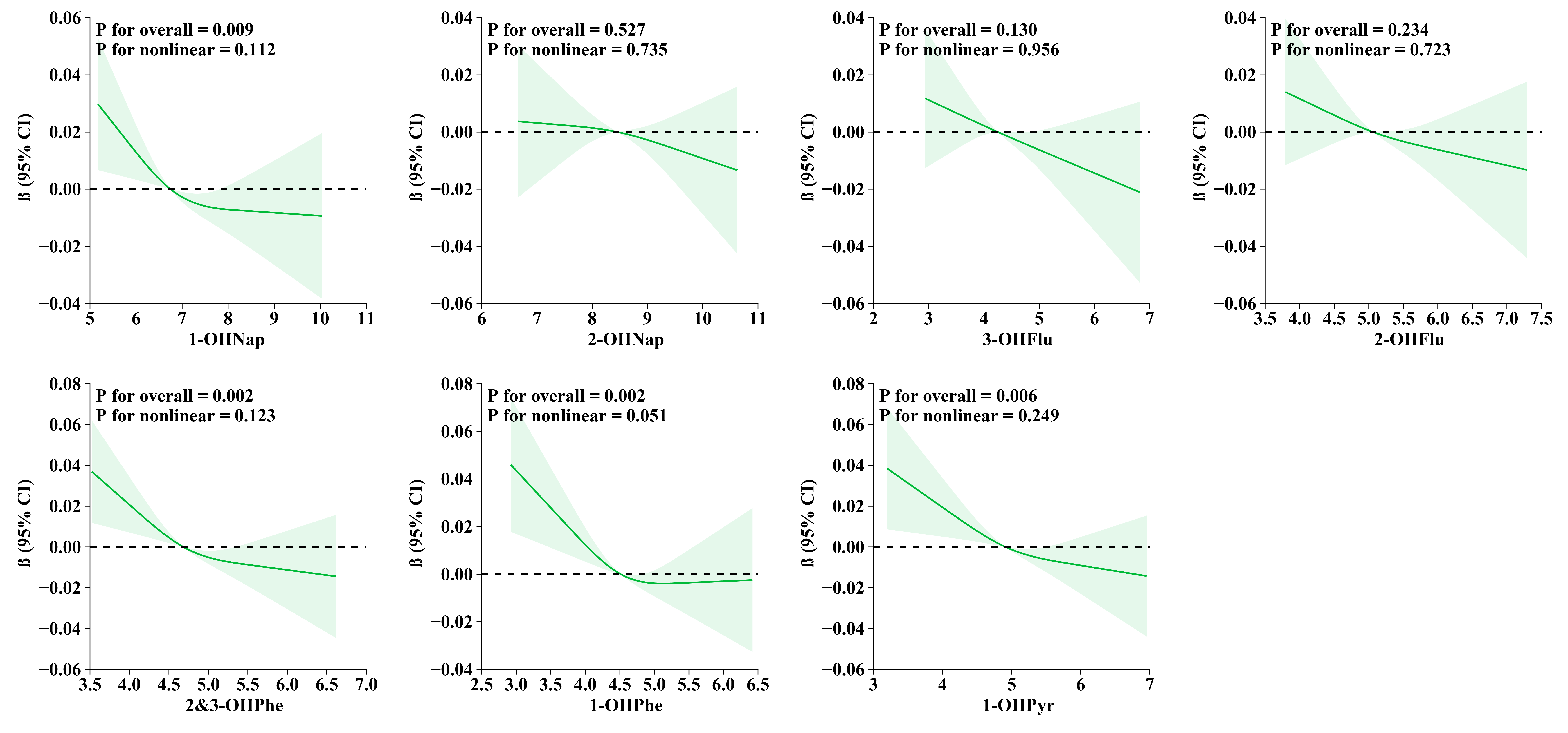


**Fig. S2** Associations of urinary OH-PAHs with lumbar spine BMD based on the restricted cubic spline models after adjusted for gender, age, race, poverty income ratio, education level, BMI, cotinine, and daily protein, calcium, and phosphorus intake. Abbreviations: OH-PAHs, Hydroxy polycyclic aromatic hydrocarbons; BMD, Bone mineral density; BMI, Body mass index.


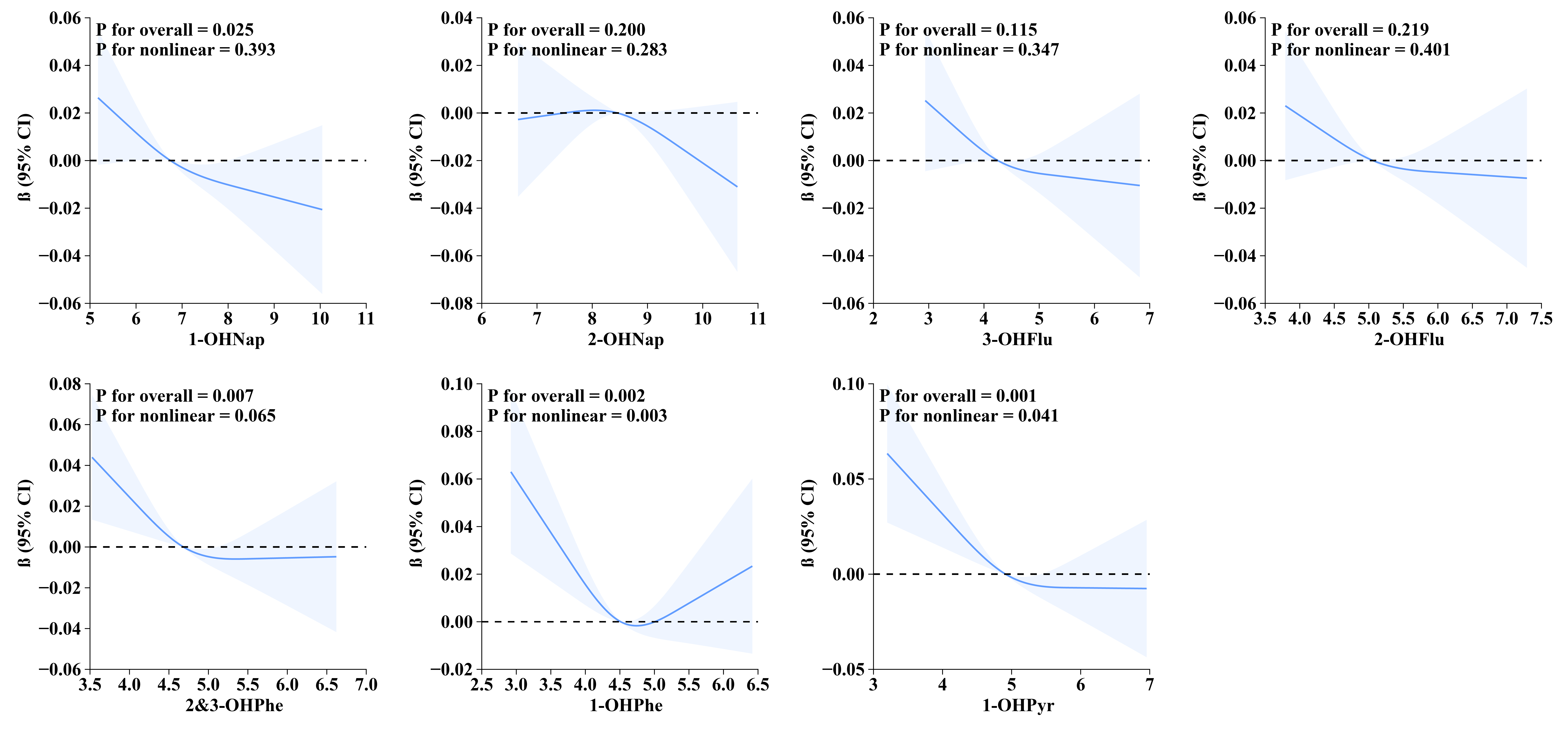


**Fig. S3** Associations of urinary OH-PAHs with pelvis BMD based on the restricted cubic spline models after adjusted for gender, age, race, poverty income ratio, education level, BMI, cotinine, and daily protein, calcium, and phosphorus intake. Abbreviations: OH-PAHs, Hydroxy polycyclic aromatic hydrocarbons; BMD, Bone mineral density; BMI, Body mass index.


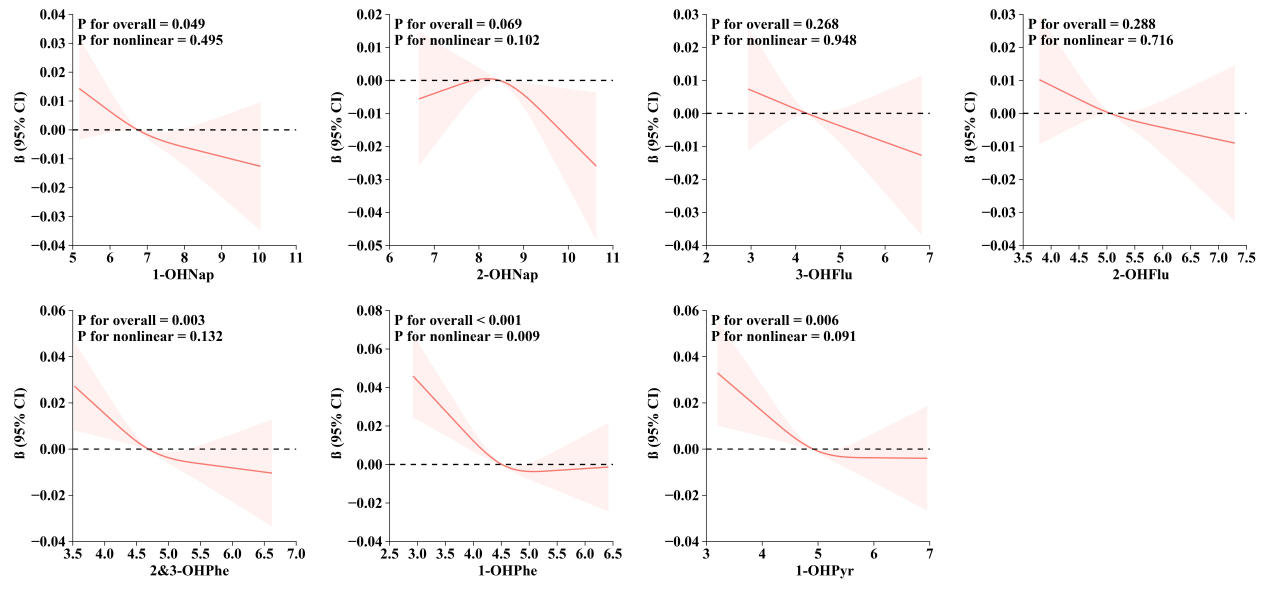


**Fig. S4** Associations of urinary OH-PAHs with total BMD based on the restricted cubic spline models after adjusted for gender, age, race, poverty income ratio, education level, BMI, cotinine, and daily protein, calcium, and phosphorus intake. Abbreviations: OH-PAHs, Hydroxy polycyclic aromatic hydrocarbons; BMD, Bone mineral density; BMI, Body mass index.


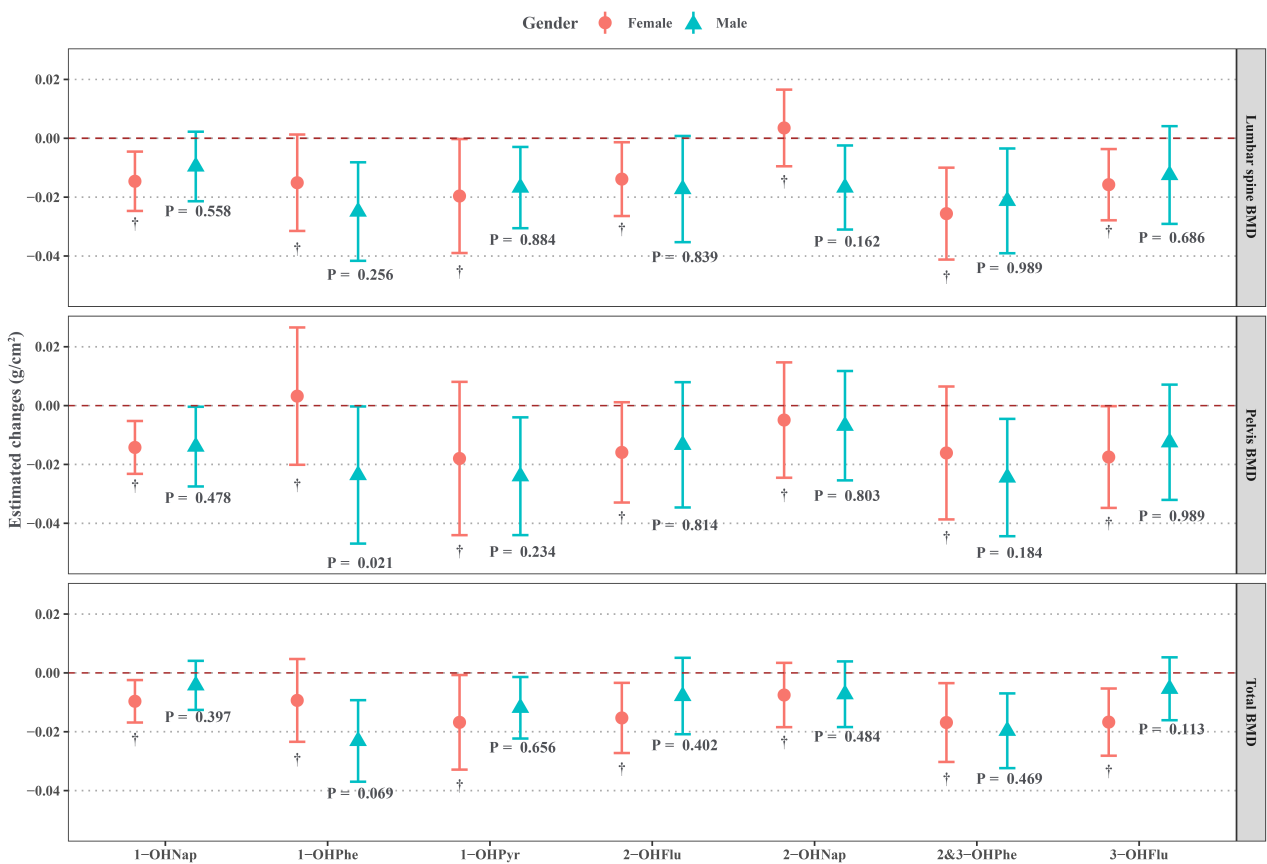


**Fig. S5** Estimated changes for associations of urinary OH-PAHs with lumbar spine BMD, pelvis BMD, and total BMD in different gender groups after adjusted for age, race, BMI, poverty income ratio, education level, cotinine, and daily protein, calcium, and phosphorus intake. Abbreviations: OH-PAHs, Hydroxy polycyclic aromatic hydrocarbons; BMD, Bone mineral density; BMI, Body mass index.


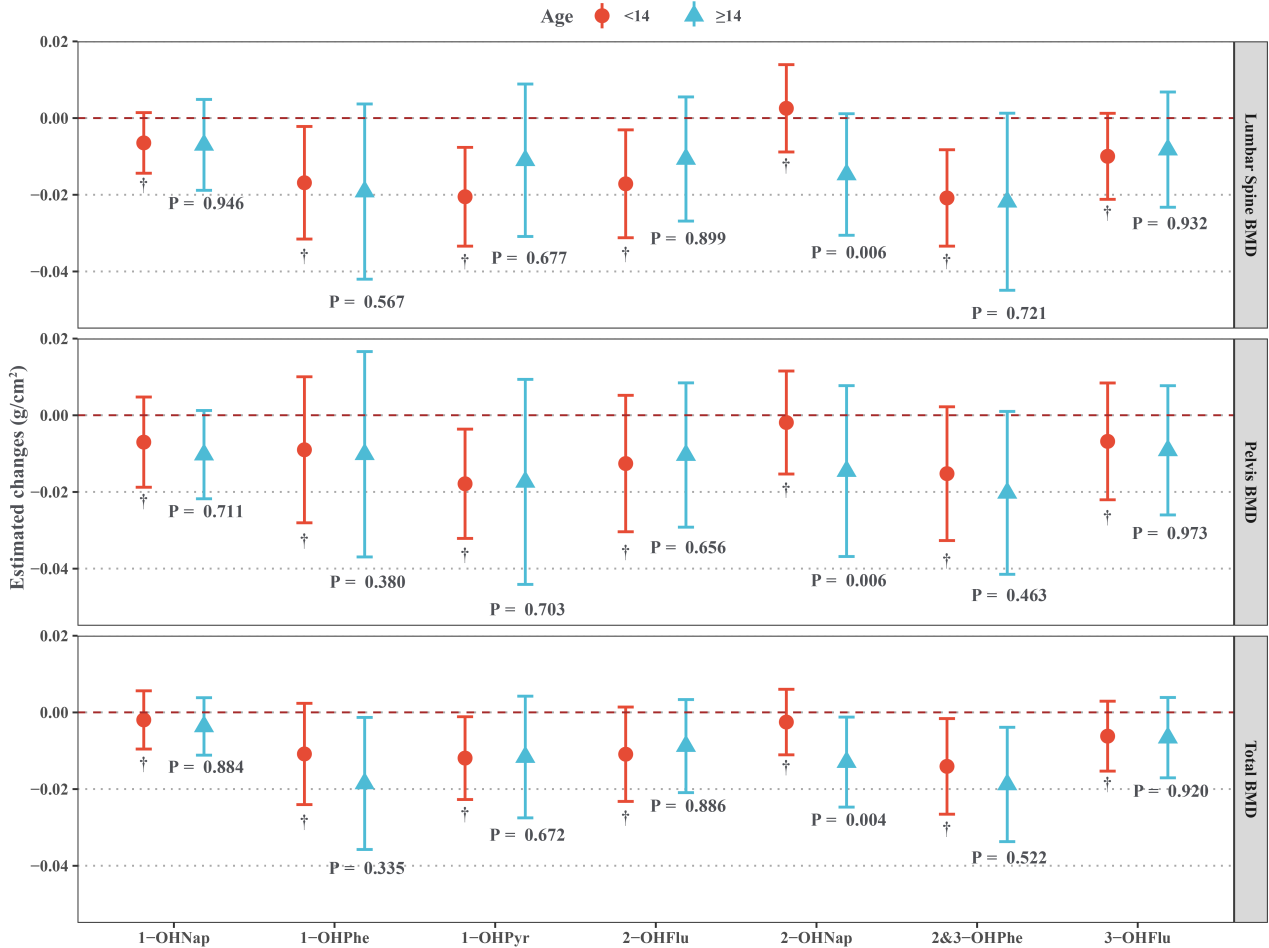


**Fig. S6** Estimated changes for associations of urinary OH-PAHs with lumbar spine BMD, pelvis BMD, and total BMD in different age groups after adjusted for gender, race, BMI, poverty income ratio, education level, cotinine, and daily protein, calcium, and phosphorus intake. Abbreviations: OH-PAHs, Hydroxy polycyclic aromatic hydrocarbons; BMD, Bone mineral density; BMI, Body mass index.


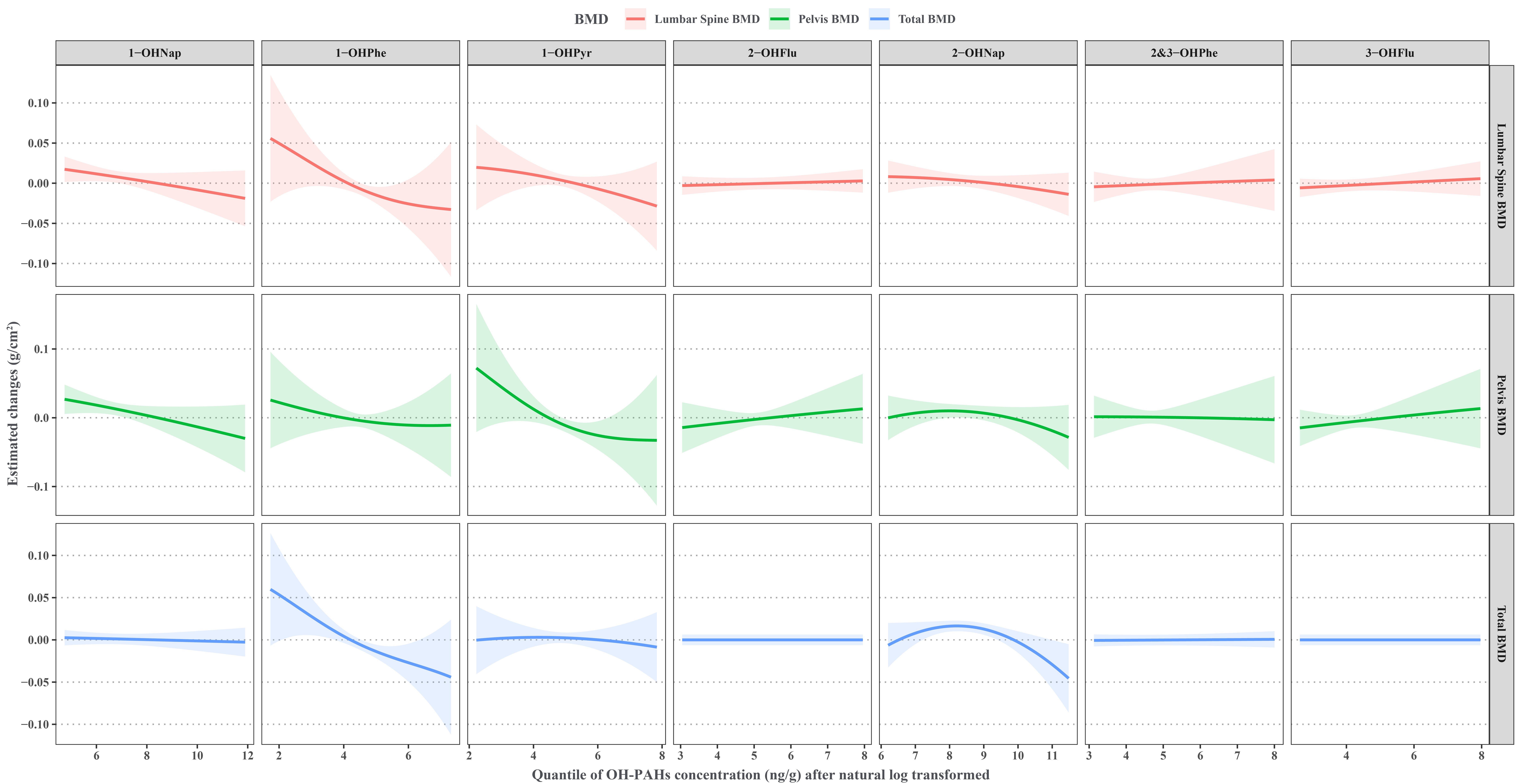


**Fig. S7** The exposure-response curve for lumbar spine BMD, pelvis BMD, and total BMD in relation to urinary OH-PAHs based on BKMR model with adjustment for gender, age, race, poverty income ratio, education level, BMI, cotinine, and daily protein, calcium, and phosphorus intake. Abbreviations: OH-PAHs, Hydroxy polycyclic aromatic hydrocarbons; BMD, Bone mineral density; BMI, BMKR, Bayesian kernel machine regression; BMI, Body mass index.

| **Table. S2** Associations of urinary OH-PAHs mixture with BMD in Qgcomp model | | |
| --- | --- | --- |
| BMD | Estimated change (g/cm^2^) ^a^ | *P* |
| Lumbar spine BMD | -0.012 (95%CI: -0.021, -0.004) | 0.006 |
| Pelvis BMD | -0.014 (95%CI: -0.025, -0.003) | 0.012 |
| Total BMD | -0.010 (95%CI: -0.016, -0.003) | 0.003 |
| Abbreviations: OH-PAHs, Hydroxy polycyclic aromatic hydrocarbons; BMD, Bone mineral density; Qgcomp, quantile g-computation; CI, Confidence interval.  ^a^ Per quartile level increase of urinary metals mixture. | | |
| 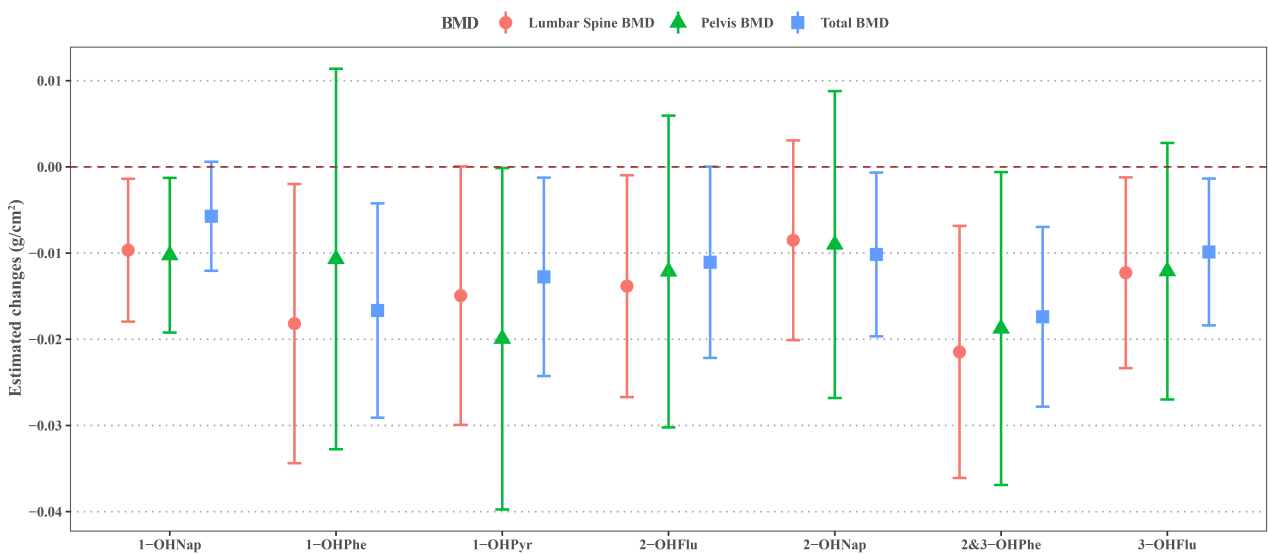  **Fig. S8** Estimated changes for associations of urinary OH-PAHs with lumbar spine BMD, pelvis BMD, and total BMD among subjects with urinary creatinine values between 30–300 mg/dL. Abbreviations: OH-PAHs, Hydroxy polycyclic aromatic hydrocarbons; BMD, Bone mineral density. | | |


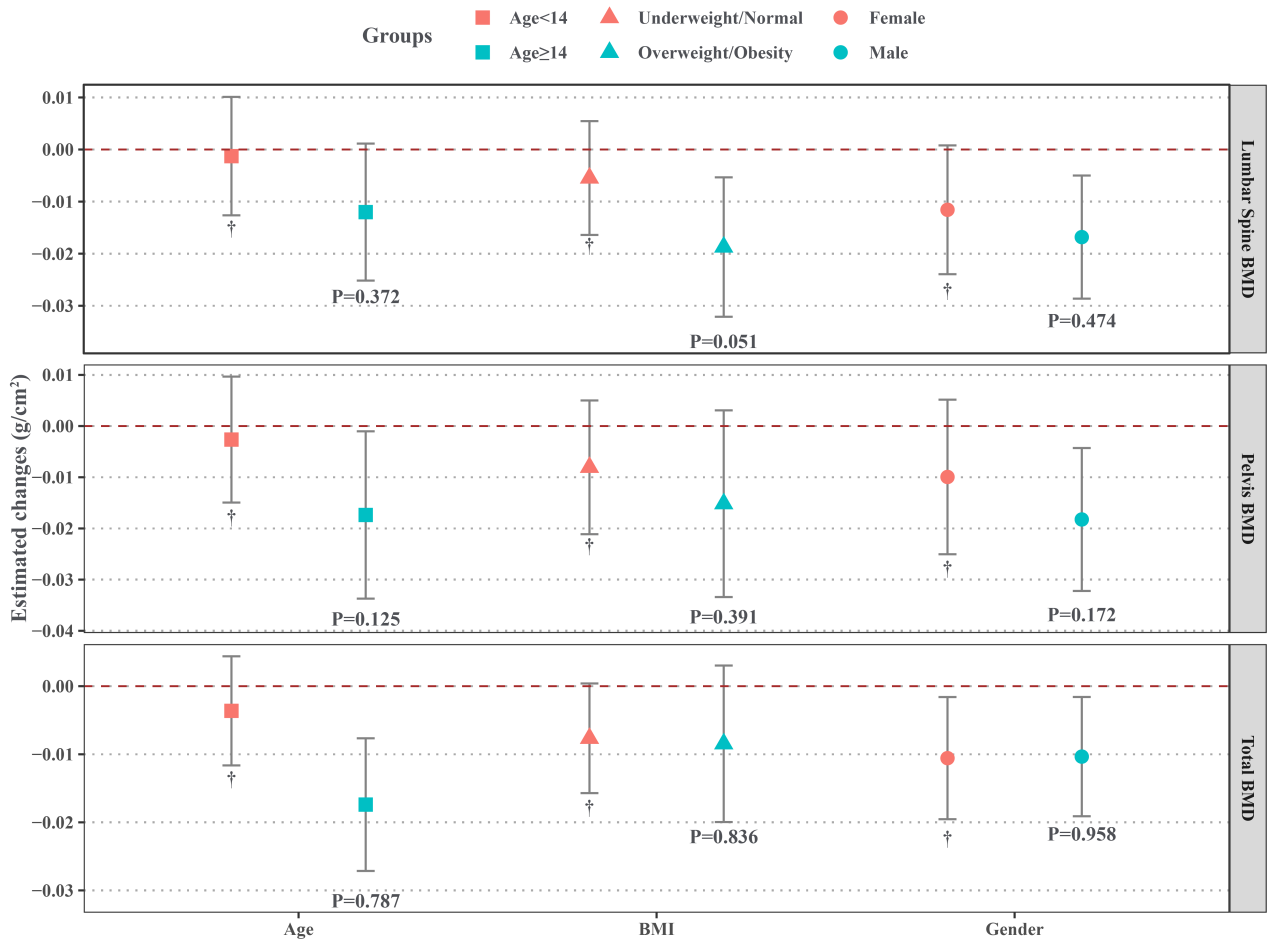


**Fig. S9** Estimated changes for associations of urinary OH-PAHs mixture with lumbar spine BMD, pelvis BMD, and total BMD in different groups based on Qgcomp model. Abbreviations: OH-PAHs, Hydroxy polycyclic aromatic hydrocarbons; BMD, Bone mineral density; Qgcomp, quantile g-computation.

| **Table. S3** Associations of urinary 1-OHPyr with BMD after excluding subjects below the LOD | | |
| --- | --- | --- |
| BMD | Estimated change (g/cm^2^) ^a^ | *P* |
| Lumbar spine BMD | -0.017 (95%CI: -0.003, -0.031) | 0.021 |
| Pelvis BMD | -0.021 (95%CI: -0.003, -0.040) | 0.030 |
| Total BMD | -0.013 (95%CI: -0.002, -0.024) | 0.023 |
| Abbreviations: 1-OHPyr, 1-Hydroxypyrene; BMD, Bone mineral density; LOD, Limit of detection CI, Confidence interval. | | |
